# Thomas Webster

**Published:** 1910-03

**Authors:** 


### THOMAS WEBSTER, M.R.C.S.Eng., L.R.C.P.Lond.

MR. THOMAS WEBSTER died at his residence at Redland on February 12th, at the advanced age of 91. He took his M.R.C.S.Eng. in 1858, and L.R.C.P.Lond. in 1866. He was Honorary Surgeon to the Redland Dispensary for a great many years. He formerly held the post of Surgeon to the Bristol Hospital for Diseases of Women and Children. Some years ago he published a report on the "Administration of the Paris Institution for Deaf and Dumb."

At the time of his death Mr. Webster was the oldest medical man in Bristol.

